# Spatiotemporal distribution of paralytic shellfish poisoning (PSP) toxins in shellfish from Argentine Patagonian coast

**DOI:** 10.1016/j.heliyon.2019.e01979

**Published:** 2019-06-20

**Authors:** Leilén Gracia Villalobos, Norma H. Santinelli, Alicia V. Sastre, Germán Marino, Gastón O. Almandoz

**Affiliations:** a Centro para el Estudio de Sistemas Marinos (CESIMAR-CONICET), Boulevard Brown 2915 (U9120ACD), Puerto Madryn, Argentina; b Instituto de Investigación de Hidrobiología, Facultad de Ciencias Naturales y Ciencias de la Salud, Universidad Nacional de la Patagonia San Juan Bosco, Gales 48 (U9100CKN), Trelew, Argentina; c Dirección Provincial de Salud Ambiental, Ministerio de Salud de la Provincia del Chubut, Ricardo Berwin 226 (U9100CXF), Trelew, Argentina; d División Ficología, Facultad de Ciencias Naturales y Museo, Universidad Nacional de La Plata, Paseo del Bosque s/n (B1900FWA), La Plata, Argentina; e Consejo Nacional de Investigaciones Científicas y Técnicas (CONICET), Av. Rivadavia 1917 (C1033AAV), Buenos Aires, Argentina

**Keywords:** Environmental science, Aquatic ecology, Marine biology, Environmental risk assessment, Environmental toxicology, Toxicology, *Alexandrium catenella*, Harmful algal blooms, Monitoring programmes, Bivalves, Mouse bioassays

## Abstract

Harmful algal blooms (HABs) have been recorded in the Chubut Province, Argentina, since 1980, mainly associated with the occurrence of paralytic shellfish poisoning (PSP) toxins produced by dinoflagellates of the genus *Alexandrium*. PSP events in this area impact on fisheries management and are also responsible for severe human intoxications by contaminated shellfish. Within the framework of a HAB monitoring program carried out at several coastal sites along the Chubut Province, we analyzed spatiotemporal patterns of PSP toxicity in shellfish during 2000–2011. The highest frequency of mouse bioassays exceeding the regulatory limit for human consumption was detected in spring and summer, with average values of up to ≈70% and 50%, respectively. By contrast, a lower percentage of positive bioassays (2–8%) or no toxicity at all was usually detected during autumn and winter. The most intense PSP events were usually observed between November and January, with values of up to 4,000 μg STX eq 100 g^−1^, and showed a marked interannual variability both in their magnitude and location. In addition, a severe PSP outbreak was recorded during autumn, 2009, at Camarones Bay, with toxicity values of up to 14,000 μg STX eq 100 g^−1^. The scallop *Aequipecten tehuelchus* showed significantly higher toxicity values compared to other shellfish species in SJG and SMG, suggesting a lower detoxification capacity. Our results contribute to the understanding of HABs dynamics on the Argentine Patagonian coast.

## 1 Introduction

Public health problems arising directly or indirectly as a result of harmful algal blooms (HABs) are of widespread occurrence. Paralytic shellfish poisoning (PSP) toxins are part of a family of highly potent neurotoxins (i.e. saxitoxin and analogs) mainly produced by dinoflagellates of the genera *Alexandrium*, *Gymnodinium*, and *Pyrodinium* (Etheridge, 2010). The impacts of PSP outbreaks include human intoxications and death from contaminated shellfish or fish, loss of wild and cultured seafood resources, impairment of tourism and recreational activities, alterations of marine trophic structure, and death of marine mammals, fish, and seabirds (Anderson et al., 2012).

The symptoms in humans range from altered perception (burning or tingling sensation and numbness of the lips, which can spread to the face and neck), headache, dizziness and nausea, to more severe symptoms such as incoherent speech, a progression of altered perception to the arms and legs, a progressive loss in limb coordination and general weakness. Respiratory distress may also occur, as a consequence of muscular paralysis progressing through the whole body, and death may be the outcome of PSP by respiratory paralysis (Rossini and Hess, 2010).

Marine bivalves, such as mussels, clams, oysters and scallops, accumulate PSP toxins in their tissues, which makes them potentially toxic to vertebrates, including humans (Estrada et al., 2007). The bioaccumulation in filter-feeding bivalves and the subsequent transfer through the food web results in potentially fatal human illnesses, which have become a widespread public health problem especially along the Pacific and Atlantic coasts of North America, in Central and South America, the western European coasts, Japan, Southeast Asia, South Africa, New Zealand, and Australia (Kacem et al., 2015).

On the Argentine coast, the first PSP outbreak was documented in the Valdés Peninsula in 1980, associated with a bloom of the species *Alexandrium catenella* (as *A. tamarense*) (Carreto et al., 1986). Since then, blooms of *Alexandrium catenella* have been recurrently observed along the Argentine coast, causing human intoxications and death from contaminated shellfish, loss of seafood resources, and death of marine fauna (Montoya et al., 2018, and references therein). In particular, human poisonings and deaths were reported in northern Patagonia in 1985 due to consumption of contaminated bivalve molluscs in Engaño Bay (Vecchio et al., 1986; Santinelli et al., 2002), and a new PSP outbreak occurred in 1988 in Nueva Bay (Nuevo Gulf) (Esteves et al., 1992; Santinelli et al., 2002). In order to mitigate the negative effects of PSP events on human health, shellfish fisheries, aquaculture and tourism, a regular monitoring program was initiated in the Chubut Province in 2000. An intense *A. catenella* bloom was recorded in the Northern Patagonian gulfs in 2011, and then expanded southwards to Camarones Bay and San Jorge Gulf. Toxin concentrations in mussels reached 3,980 μg STX eq 100 g^−1^, causing human poisonings and one fatality in Caleta Hornos, close to Camarones Bay (Baulde, 2011), associated with harvesting in forbidden period.

The most significant fishery resource in the Chubut Province is a metapopulation of the Tehuelche scallop (*Aequipecten tehuelchus)* located in the San José gulf (Amoroso and Gagliardini, 2010; Amoroso et al., 2011). However, the ribbed mussel (*Aulacomya atra*), the blue mussel (*Mytilus platensis*) and the clam (*Ameghinomya antiqua*) also support important regional fisheries in the Northern Patagonian gulfs.

Despite the impact of PSP events on public health and fisheries in Northern Patagonia (Chubut Province), studies on the spatial and temporal patterns of shellfish toxicity are still lacking. Here, we analyze results obtained within the framework of the monitoring program along the coast of Chubut (Northern Patagonia, 42–46° S) during the period 2000–2011. We used a subset of 1,702 mouse bioassay analysis focusing on spatial and seasonal variability to interannual patterns of timescales.

## 2 Method

### 2.1 Study area description

**Fig. 1:**
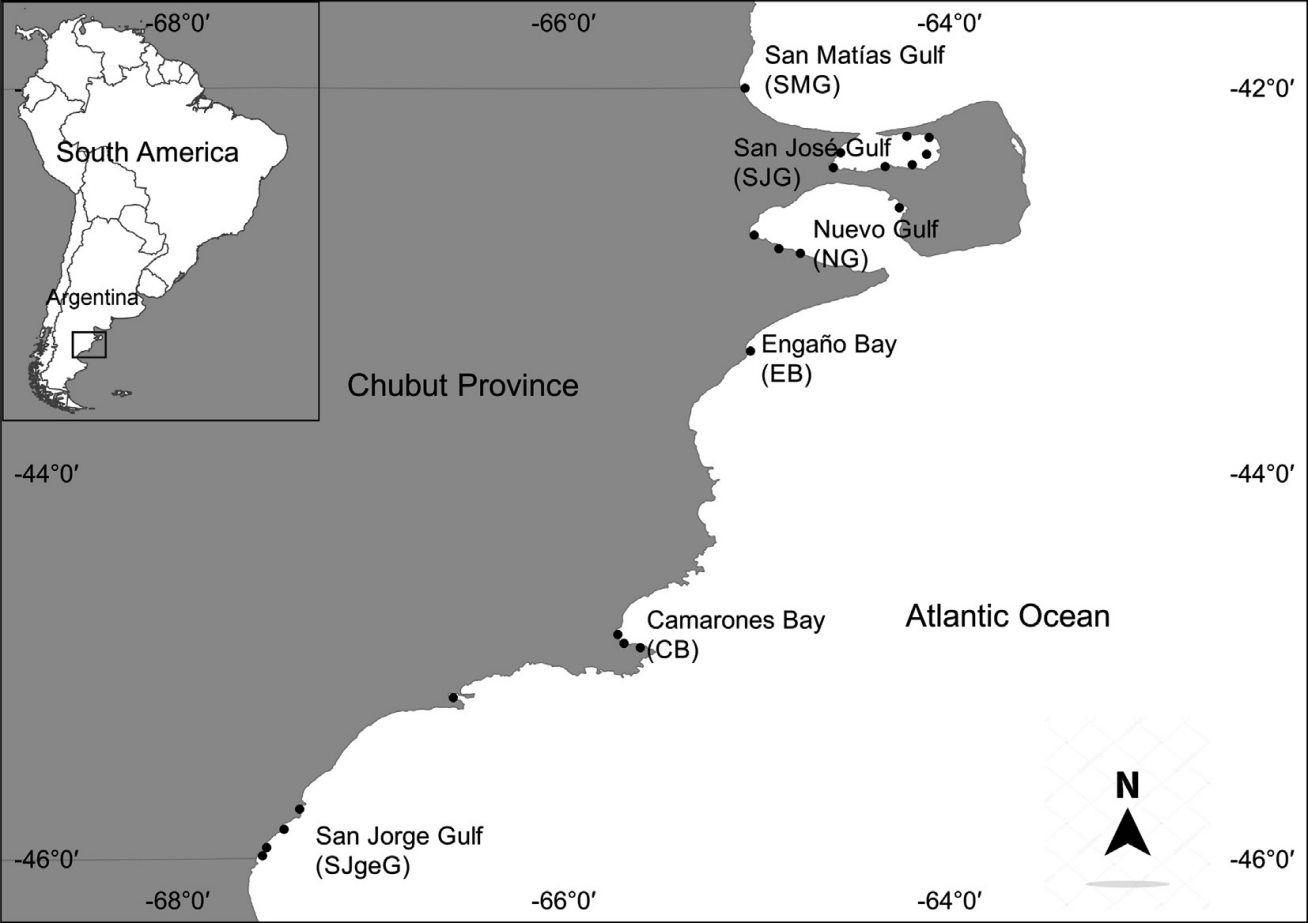
Map of the study area and location of sampling sites in Northern Patagonia, Argentina.

The study area includes the Chubut Province coast, between 42° and 46° S, i.e. Northern Patagonia, Argentina (Fig. 1). The San Matías Gulf (SMG) is the largest of the three northern Patagonian gulfs (18,000 km^2^) and presents two well defined areas separated by a frontal system (Amoroso and Gagliardini, 2010). In contrast, the San José Gulf (SJG), subelliptic in outline and semi-enclosed, is the smallest (817 km^2^) and shallowest (mean depth 30 m, maximum 80 m) of the northern Patagonian gulfs. The Nuevo Gulf (NG) is a roughly elliptical, semi-enclosed body of water (2,500 km^2^) in communication with the southwestern Atlantic Ocean through a 17 km wide strait (Rivas and Beier, 1990). The Engaño Bay (EB) is an area of exposed coast between 43° 15′ 02″ S and 43° 22′ 41″ S, spreading to the south of the Chubut River estuary. The Chubut River has an average discharge in the bay in the order of 50 m^3^ s^−1^, with maximums that can exceed 200 m^3^ s^−1^ (Commendatore et al., 1996).

Southwards, the Camarones Bay (CB) is located at 44°48′ S and covers a coastal marine area of approximately 52 km^2^. Finally, the San Jorge Gulf (SJgeG) is a semi-open basin of the Argentine Sea located approximately between 45° and 47° S that covers a total area of 39,340 km^2^ (Fernández, 2006).

### 2.2 Shellfish sampling

Shellfish toxicity data from 2000 to 2011 was acquired within the framework of the HAB and Shellfish Toxicity Monitoring Program carried out in Chubut coastal waters since the year 2000, as part of the Provincial Plan for Prevention and Control of Red Tides established by the Secretaría de Pesca.

The dataset contains a total of 1,702 toxicity records for 21 sampling sites, distributed approximately between 42°–46°S and 64°–67°30′W (Fig. 1). Sampling was carried out monthly, although there was a lower sampling frequency in the first years of monitoring (2000–2004).

Shellfish samples were collected from harvested beds and removed from subtidal sand beds and from the substrate by diving at 5–25 m of depth.

The analyzed shellfish species included Tehuelche scallops (*Aequipecten tehuelchus*), clams (*Ameghinomya antique*), mussels (*Mytilus platensis*), ribbed mussels (*Aulacomya atra*), Geoduck clams (*Panopea abbreviata*) and razor clams (*Ensis macha*). Different shellfish species were collected from each zone according to their natural distribution (Table 1). The number of shellfish collected per sample was usually ≥30 individuals of each species, with the exception of the Geoduck clam (N = 10), because of its large size and high market value. The collected shellfish were kept frozen at -20 °C until toxin analysis.

**Table 1:** Detail of sampling sites and shellfish species collected from each zone in Patagonia, Argentina.

Zones	Sites per zone	Shellfish species sampled
San Matías Gulf (SMG)	1	*A. atra* (N = 43), A. *tehuelchus* (N = 23), *P. abbreviata* (N = 67) and *E. macha* (N = 68)
San José Gulf (SJG)	10	*A. tehuelchus* (N = 403), *A. antique* (N = 234), *M. platensis* (N = 125) and *A. atra* (N = 211)
Nuevo Gulf (NG)	4	*A. atra* (N = 205)
Engaño Bay (EB)	1	*M. platensis* (N = 74)
Camarones Bay (CB)	1	*M. platensis* (N = 75)
San Jorge Gulf (SJgeG)	4	*M. platensis* (N = 174)

### 2.3 Mouse bioassays

Toxin levels were acquired using the standardized mouse bioassay (MBA) at the Dirección Provincial de Salud Ambiental (DPSA) of Chubut Province, and expressed in saxitoxin equivalents (STX eq). The MBA method was originally developed by Sommer and Meyer (1937), later standardized and validated through successive interlaboratory studies, and nowadays is still considered the official regulatory method in several countries for determining PSP toxins in shellfish (AOAC, 1990, 2005; Fernández et al., 2002). Briefly, twenty gram mice are injected with 1 ml of an acid extract of the shellfish and the time taken for the animal to die is recorded. Highly toxic extracts are diluted to ensure that mortality occurs within 5–7 minutes (AOAC, 2005). Toxicity is then calculated in reference to dose response curves established with STX standards, and expressed in mouse units (MU). One MU is the amount of injected toxin which would kill a 20 g mouse in 15 minutes, and is equivalent to 0.18 μg of STX. At present, harvesting sites in Argentina are closed whenever the toxin concentrations in shellfish reach ≥80 μg STX eq 100 g^−1^ tissue. The detection limit of the MAB is approximately 40 μg STX 100 g^−1^ of shellfish tissue with a precision of ±15–20 percent (FAO, 2004).

### 2.4 Data analysis

To analyze the seasonality and interannual variation of the occurrence of PSP outbreaks, as well as toxicity levels by species, only data from 2005-2011 was used, because of the greater sampling frequency during this period. In those areas where more than one bivalve species was sampled (i.e. SMG and SJG), only data from the most abundant species was used. For the analysis of the spatial variation in those species present in at least two areas (i.e. *A. tehuelchus*, *A. atra* and *M. platensis*) and the toxicity of the different species by area (i.e. SJG and SMG), the complete data set (2000–2011) was used.

Statistical analyses were performed using the STATISTICA 7.0 software package. The data were checked for normality and homogeneity of variance using Shapiro–Wilks and Levene's test (p < 0.05), respectively (Zar, 1999). All tested cases proved non-normal distribution. Thus, spatiotemporal distribution analysis of toxicity in shellfish species and the interspecific differences in San José and San Matías gulfs were assessed by the non-parametric Kruskal–Wallis and Mann Whitney analysis of variance, and post-hoc Tukey's range test.

The spatiotemporal distribution map of PSP outbreaks was performed using QGIS software (QGIS 1.8.0-Lisboa, 2012).

## 3 Results

### 3.1 Seasonal patterns of PSP outbreaks

**Fig. 2:**
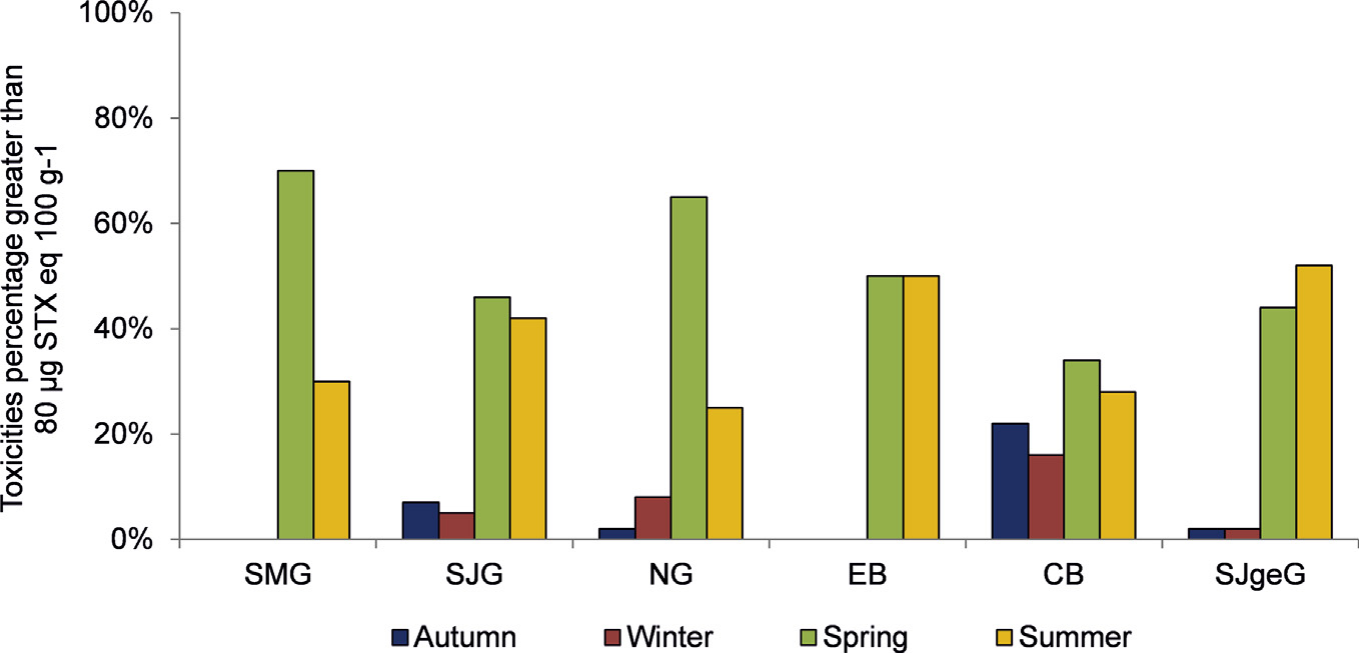
Seasonal variation of percentages of PSP mouse bioassay results that exceeded the regulatory limit for human consumption (≥80 μg STX 100 g^−1^ tissue) in the most representative shellfish species from each zone, between 2005 and 2011. SMG (N = 63); SJG (N = 303); NG (N = 167); EB (N = 61); CB (N = 72); SJgeG (N = 164).

Mouse bioassay results exceeding the regulatory limit for human consumption, i.e. 80 μg STX eq 100 g^−1^ tissue, were mostly detected during spring and summer in all areas (Fig. 2). By contrast, only a low percentage of positive mouse bioassays (2–8%) or no toxicity at all were detected during autumn and winter, with the exception of high values observed in CB (22% and 16%, respectively). Particularly, the percentages of positive mouse bioassays detected in spring duplicated those observed in summer in SMG and NG.

**Fig. 3:**
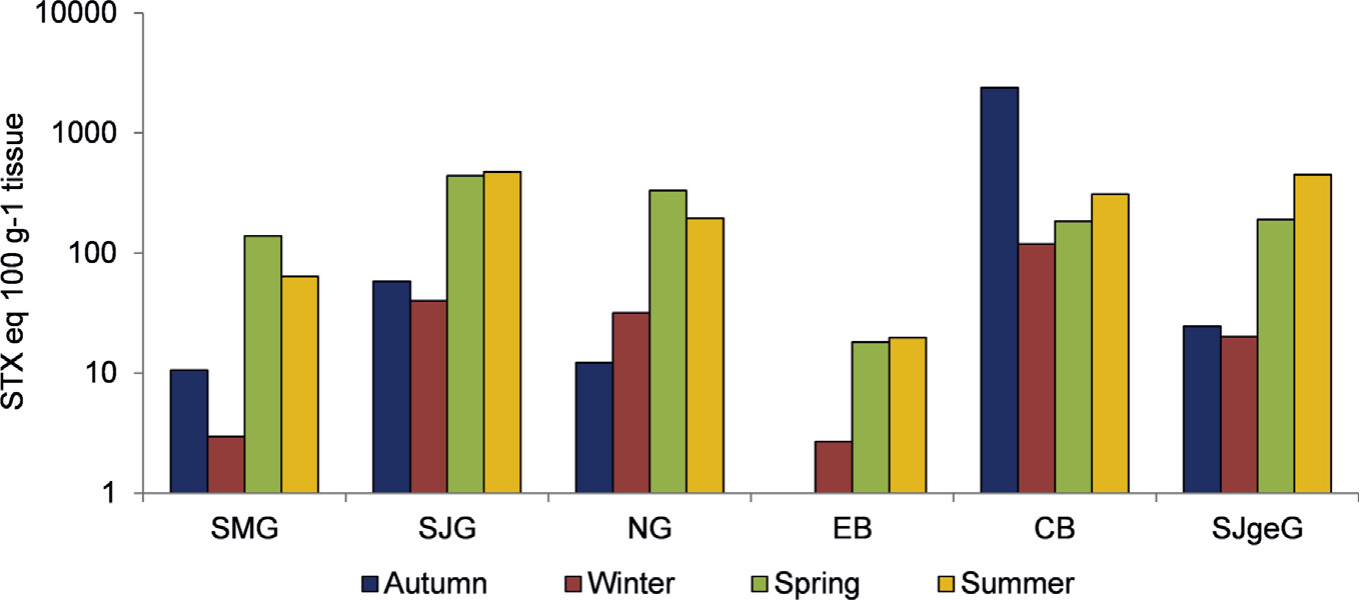
Seasonal variation of mean mouse bioassay toxicity values, observed in the most representative shellfish species from each zone, between 2005 and 2011. The y axis is represented in a logarithmic scale.

Mean toxicity values, for each area and season of the year, were also higher in spring and summer, with the exception of CB, where the mean was higher in autumn (Fig. 3). In CB and SJgeG, mean toxicity values were higher in summer than in spring, contrary to that observed in SMG and NG, whereas means values in SJG and EB were similar in both seasons.

### 3.2 Interannual variation of PSP outbreaks during 2005–2011

**Fig. 4:**
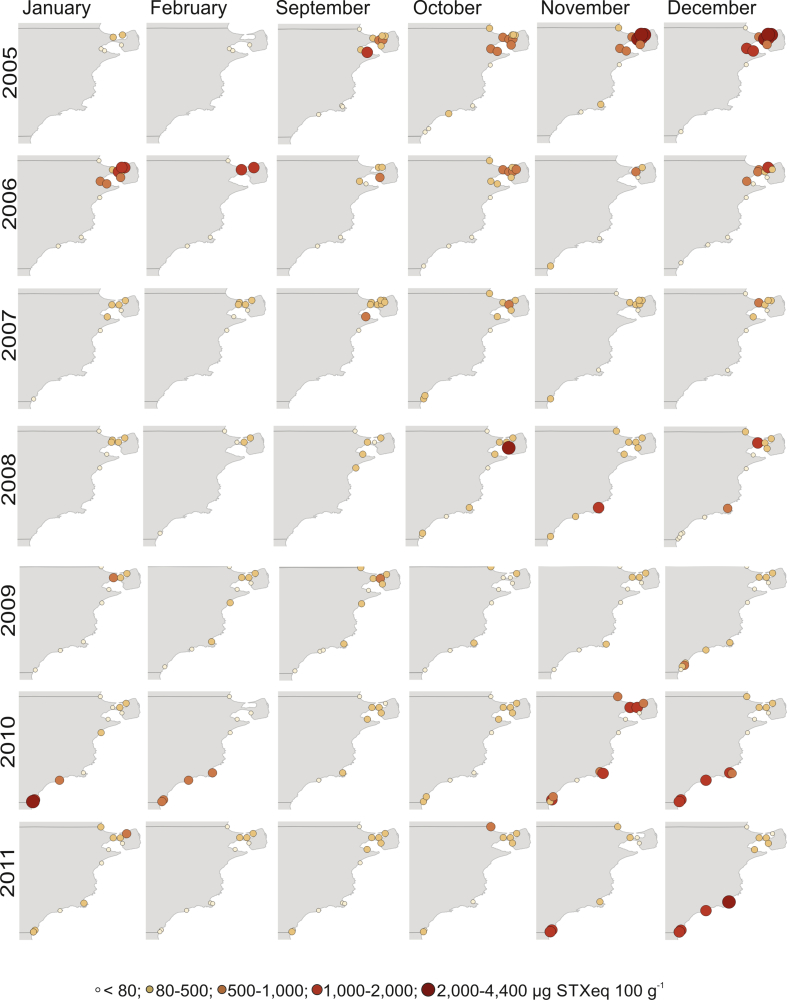
Values of PSP toxins detected in shellfish by mouse bioassay during spring and summer 2005–2011. The PSP values are expressed in μg STX 100 g^−1^.

During 2005–2007, the most intense PSP events were recurrently observed in SJG, i.e. the northern zone, while values usually lower than 80 μg STX eq 100 g^−1^ and exceptionally between 80 and 500 μg STX eq 100 g^−1^, were registered in the southern sites (Fig. 4). The highest toxicity values reached in each year were ≈4,000 μg STX eq 100 g^−1^ (November, 2005), ≈1,900 μg STX eq 100 g^−1^ (January, 2006), and ≈900 μg STX eq 100 g^−1^ (December, 2007). Mean spring-summer toxicity values observed in SJG during 2005 were almost twice as high as those detected in 2006 and three times higher than those detected in 2007.

During spring-summer 2008–2009, moderate PSP events were usually observed throughout the study area, with the exception of a peak of ≈2,000 μg STX eq 100 g^−1^ in NG (October, 2008). The highest toxicity value during 2009 was observed in SJG (≈700 μg STX eq 100 g^−1^, January, 2009). Despite the relatively low toxicity values observed during spring-summer 2009, a severe PSP outbreak was recorded during April 2009 (autumn) in CB, with toxicity values in *M. platensis* of up to 14,000 μg STX eq 100 g^−1^.

During 2010–2011, major PSP outbreaks occurred mainly in the southern area. The highest toxicity values were observed in SJgeG and CB, reaching values of up to 2,200 μg STX eq 100 g^−1^ (January, 2010) and 4,000 μg STX eq 100 g^−1^ (December, 2011), respectively.

### 3.3 Spatial patterns of toxicity in selected shellfish species

Toxicity levels in *A. atra* exceeded 80 μg STX eq 100 g^−1^ in 39% of the analyzed samples from SJG, while in SMG and NG percentages were lower (between 25 and 28%). The maximum toxicity values recorded for this species were in a range between 1,500 and 3,300 μg STX eq 100 g^−1^ in the three study areas (Table 2), although no significant differences between them were found (Kruskal-Wallis test, p = 0.3058).

**Table 2:** Percentages of PSP observations exceeding the regulatory limit for human consumption and maximum toxicity values detected in the main shellfish species from the Argentine Sea.

Species/Zone		% PSP ≥80 μg	Maximum toxicity	Month/Year
		STX eq 100 g^−1^	(μg STX eq 100 g^−1^)	
*A. atra*	SMG	28	1,524	October, 2011
	SJG	39	3,341	November, 2005
	NG	25	2,004	October, 2008
*A. tehuelchus*	SMG	35	449	April, 2011
	SJG	51	4,111	November, 2005
*M. platensis*	SJG	24	1,483	October, 2010
	EB	12	2,343	November, 2000
	CB	43	14,946	April, 2009
	SJgeG	26	2,223	January, 2010

*A. tehuelchus* showed high toxicity percentages above 80 μg STX eq 100 g^−1^ in SJG (51%) and, to a lesser extent, in SMG (35%) (Table 2). Toxicity values in this species exhibited significant differences between both areas (Mann-Whitney U test, p = 0.0158). The maximum toxicity peak detected in SJG was one order of magnitude higher than that recorded in SMG (4,111 and 449 μg STX eq 100 g^−1^, respectively) (Table 2).

The species *M. platensis* showed a marked variability in toxicity percentages above the limit for human consumption (80 μg STX eq 100 g^−1^) in the four areas in which it was detected. The highest value was registered in CB (43%), the lowest percentage was recorded in EB (12%), while intermediate values were observed in the other two areas, SJG and SJgeG (24 and 26%, respectively). The maximum toxicity values recorded for this species were in a range between 1,400 and 14,000 μg STX eq 100 g^−1^, but no significant differences were found between areas (Kruskal-Wallis test, p = 0.1562). It should be noted that the toxicity peak registered for *M. platensis* in CB (14,946 μg STX eq 100 g^−1^) represented the highest PSP value for the entire study period.

### 3.4 Interspecific variability in SJG

Inside SJG, two sites where several species of bivalves coexist were analyzed. Site 1 is located to the south of SJG (Larralde Beach) and includes the species *A. atra*, *A. tehuelchus* and *A. antique*, while site 2 is located to the southwest of SJG (Riacho San José) and includes *M. platensis*, in addition to the aforementioned species.

**Fig. 5:**
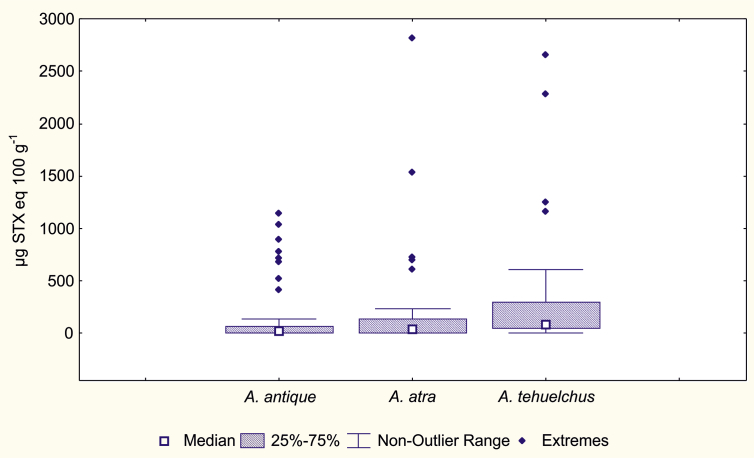
Toxicity variation between shellfish species in site 1 of SJG.

**Fig. 6:**
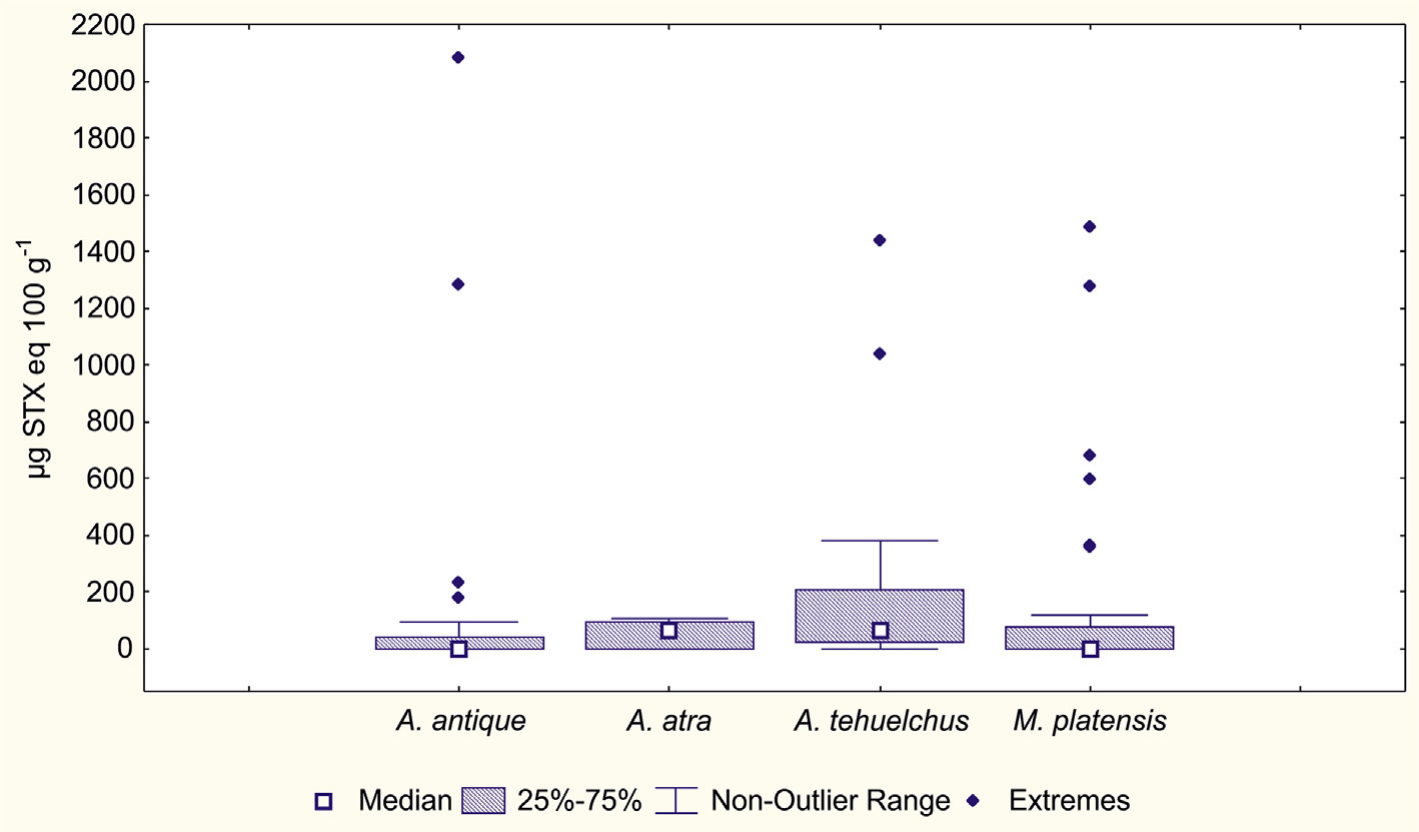
Toxicity variation between shellfish species in site 2 of SJG.

Recorded PSP toxin values for the different species of bivalve molluscs presented significant differences both in site 1 and site 2 (Kruskal-Wallis p = 0.0001 and p = 0.0002, respectively). In site 1, toxicity in *A. tehuelchus* was significantly higher than in *A. antique* and *A. atra*, while there was no difference between the last two species (Fig. 5). In site 2 of SJG, PSP values in *A. tehuelchus* differed significantly from the values in *A. antique* and *M. platensis*, but not from those in *A. atra*. On the other hand, in site 1, the maximum PSP value was registered in *A. atra* (2,800 μg STX eq 100 g^−1^), while in site 2 the maximum value was recorded in *A. antique* (2,078 μg STX eq 100 g^−1^) (Figs. 5 and 6).

### 3.5 Interspecific variability in SMG

PSP toxin values for the four species of molluscs present in SMG, i.e. *A. atra, A. tehuelchus, E. macha* and *P. abbreviata*, showed significant differences (Kruskal-Wallis, p = 0.0040), among which *A. tehuelchus* differed significantly from *E. macha*.

**Fig. 7:**
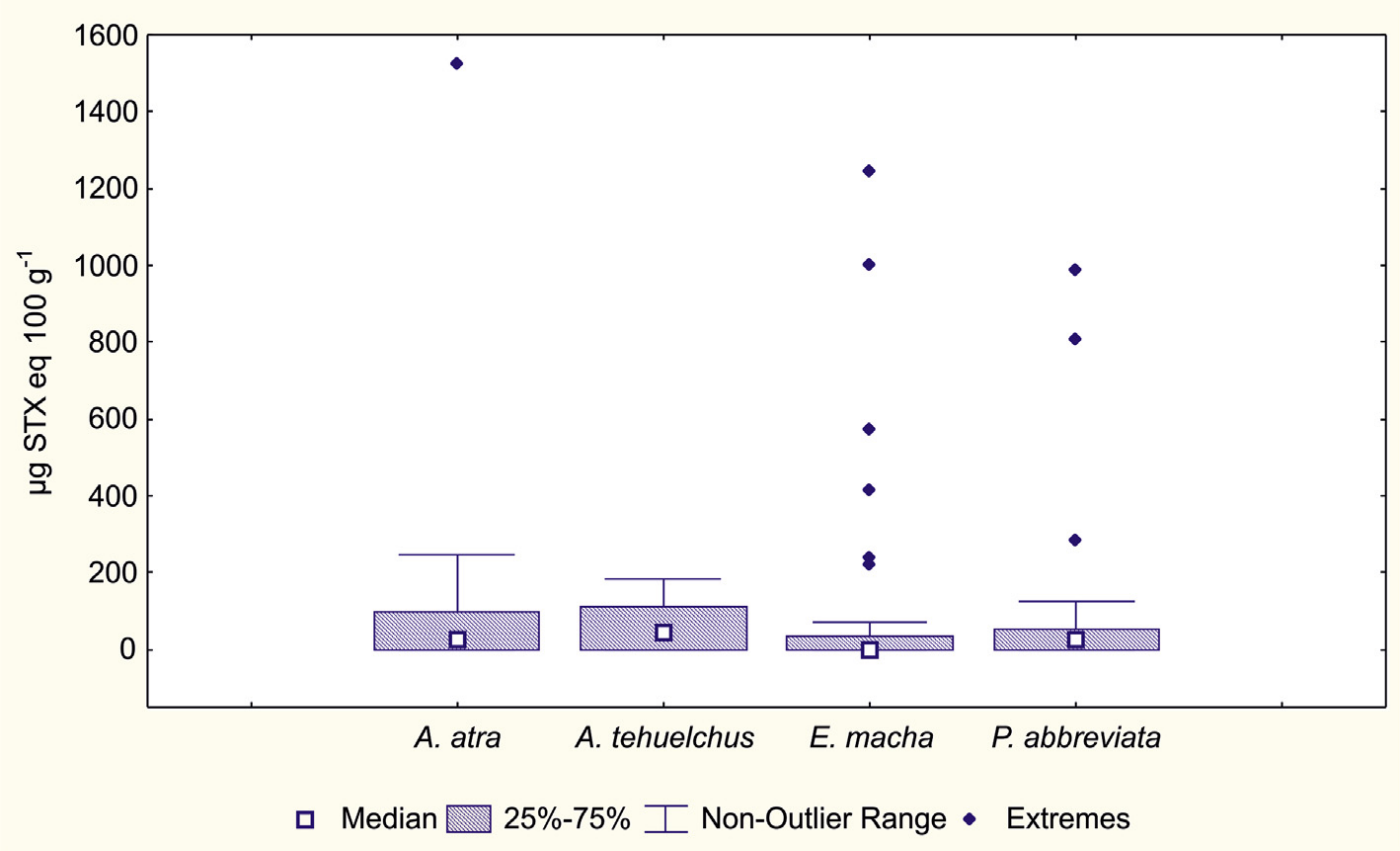
Toxicity variation between shellfish species in SMG.

The maximum PSP value was registered in *A. atra* (1,524 μg STX eq 100 g^−1^). In contrast, *A. tehuelchus* presented moderate toxicity values (maximum = 450 μg STX eq 100 g^−1^) (Fig. 7).

## 4 Discussion

In the Southwest Atlantic, blooms of the dinoflagellate *A. catenella* have been detected since 1980 along coastal areas between 37°S (Province of Buenos Aires) and 55°S (Beagle Channel) (Carreto et al., 1981, 1998; Esteves et al., 1992; Benavides et al., 1995; Akselman, 1996; Gayoso and Fulco, 2006; Montoya et al., 2010; Krock et al., 2015; Fabro et al., 2017; Krock et al., 2018). In particular, toxigenic blooms of *A. catenella* are widespread throughout all monitored sites on the coast of the Chubut Province (42–46°S) (Sastre et al., 2018). In accordance with these previous results, our study shows for the first time the widespread and frequent occurrence of PSP toxins in different shellfish species along the northern Argentine Patagonian coast during a long time period.

Our results showed that toxicity in shellfish species was more frequent and of a greater magnitude in spring and summer in all examined areas. This is in accordance with Carreto et al. (1998) who observed the greatest toxicity values in mussels beds in late spring/summer in the Patagonia region, associated with *A. catenella* blooms. In addition, our results also agree with those of Sastre et al. (2018) who found that the dinoflagellate *A. catenella* produces recurrent toxic events during spring and summer in the Chubut Province. By contrast, the maximum toxicity detected in our study (14,000 μg STX eq 100 g^−1^, in CB in 2009) was observed in autumn, which is outside of the typical annual bloom period for toxin accumulation in shellfish (Carreto et al., 1998; Sastre et al., 2018). Chlorophyll-a (Chl-a) and sea surface temperature (SST) satellite data (MODIS-Aqua-Giovanni-https://giovanni.gsfc.nasa.gov/giovanni/) for that period showed that in March 2009 Chl-a concentrations in CB were higher (∼4 mg m^−3^) than the climatological average of the period 2003–2017 (∼2 mg m^−3^) and the SST was lower (13.5 °C) than the climatological average for the same period (14 °C). We infer that the low water temperatures in CB during autumn 2009 could have favored the development of *A. catenella* blooms and the subsequent PSP outbreaks. Previous studies suggested that low temperature and nutrient rich waters promote blooms of *A. catenella* in the Argentine Sea (Carreto et al., 1986; Montoya et al., 2010; Fabro et al., 2018). In addition, Krock et al. (2015) found high dinoflagellate abundances in autumn 2012 in the SJgeG region, mainly dominated by *Ceratium* species but also including toxigenic species, such as *A. catenella* and *Protoceratium reticulatum*.

We found a marked interannual variability both in the magnitude and location of the main PSP events observed in this study. The interannual variability in PSP events agree with the differential development and magnitude of blooms of *A. catenella* reported in coastal waters of the Chubut Province by Sastre et al. (2018). In addition, these authors mention that moderate densities of *A. catenella* (i.e. 10^4^ cells L^−1^) are usually enough to cause toxic events in the northern areas, while more intense blooms of *A. catenella* (≈10^6^ cells L^−1^) are usually necessary to produce toxin accumulation in shellfish in the southern areas. As it is well known, environmental parameters such as irradiance, temperature, salinity and inorganic nutrients affect toxin content and composition of *Alexandrium* strains (Anderson et al., 2012). Thus, a potential reason for the differences in regional toxicity could be linked to strain-specific responses to environmental conditions (Montoya et al., 2018). In agreement with this, Montoya et al. (2010) have shown that the variability in toxin content and composition of *A. catenella* populations in the Argentine Sea were correlated with in situ temperature and nitrate concentration. In Argentine Patagonia, Sastre et al. (2018) reported that the monthly mean nitrate concentration in the southern area was twice as high as that in the northern zone (10.5 μM and 5 μM, respectively), and the sea temperature in the northern area was in a range of between 10.7 °C and 17.5 °C, while in the southern zone it ranged between 8.4 °C and 15.5 °C. On the other hand, Fabro et al. (2017) reported the presence of the potential PSP producer species *A. ostenfeldii* and *A. minutum* in the Argentine Sea (≈39–47°S), although they were mainly found in slope waters. The production of PSP toxins was later confirmed in three *A. ostenfeldii* strains isolated from middle-shelf waters around 41°S (Guinder et al., 2018), suggesting these species could also play a significant role in PSP events in the Argentine Sea.

Mouse bioassay maximum toxicity values detected in our study (between 449 and 14,946 μg STX eq 100 g^−1^, Table 2) are comparable with PSP concentrations associated with *Alexandrium* blooms in the Bay of Fundy, Canada, with values of up to 9,100 μg STX eq 100 g^−1^ in *Mya arenaria* (Martin et al., 2014)*.* By contrast, Molinet et al. (2010) recorded ≈30,000 μg STX eq 100 g^−1^ in *A. atra* and *M. chilensis* in the Aysén Region, in Southern Chile, associated with *A. catenella* blooms. In Argentine Patagonia, previous PSP concentrations reported by Andrinolo et al. (1999) in shellfish from Nuevo Gulf (max. of 631 μg STX eq 100 g^−1^ in *A. atra*), were much lower than those found in our study. However, exceptional records up to 50,000 μg STX eq 100 g^−1^ in mussel beds (*Mytilus edulis*) collected in the frontal region of the Valdés Peninsula were reported by Carreto et al. (1981), which shows that the toxicity in shellfish species in the Argentine Patagonia region is highly variable.

The species *A. tehuelchus*, which is the most important fishing resource in the Chubut Province, showed significant differences in toxicity values with respect to other shellfish species in SJG and SMG. In addition, *A. tehuelchus* presented a higher toxicity in SJG than in SMG, while *A. atra* and *M. platensis* did not show significant differences in toxicity between zones. Several studies reported the existence of marked differences in the toxin accumulation process among bivalve species, as a result of species-specific variation in their sensitivity to toxins (Shumway and Cucci, 1987; Lesser and Shumway, 1993; Oshima, 1995; Bricelj and Shumway, 1998; Bricelj et al., 2005). In particular, scallops such as *A. tehuelchus* have marked differences in the rate of accumulation and elimination of toxins with respect to other species of bivalves (Oshima et al., 1982), and show a lower detoxification rate than *A. atra* (Carreto et al., 1981; Santinelli, 2013). Shumway (1990) reported that mussels of the genus *Mytilus* accumulate PSP toxins more rapidly than other groups of bivalves. They also appear to eliminate the toxins in a shorter period of time (Velásquez and Navarro, 2014). This is in contrast with observations in the clam *Mercenaria mercenaria* (Bricelj et al., 1991), the scallop *Placopecten magellanicus* (Shumway et al., 1988) and the oyster *Crassostrea gigas* (Bougrier et al., 2003), all of them characterized by slow accumulation of toxins in their tissues. Moreover, a direct comparison of digestive gland toxicities between bivalve species from Japan, revealed that the scallops (*Patinopecten yessoensis* and *Chlamys nipponensis*) became about three times more toxic than the mussel *M. edulis,* and *P. yessoensis* also maintained a higher toxicity for a longer period (Oshima et al., 1982). In addition, Cembella et al. (1993) showed that the scallop *Placopecten magellanicus* and the clam *Spisula solidissima* present differences in the accumulation-elimination of toxins due to the different intoxication and detoxification dynamics. Therefore, the higher PSP toxicity values observed in *A. tehuelchus* compared with the other shellfish species (i.e. *A. atra, A. antique, M. platensis* and *E. macha*) in SJG and SMG could be linked to its longer intoxication and depuration time. By contrast, mussels such as *A. atra* and *M. platensis* are considered rapid to moderate detoxifiers (Carreto et al., 1981; Bricelj and Shumway, 1998; Andrinolo et al., 1999).

## 5 Conclusions

The presence of PSP toxins in shellfish was frequently detected in all examined zones with natural beds in the Chubut Province, Argentina. PSP toxin values exceeding the regulatory limit for human consumption were mainly observed during spring and summer, showing geographical differences in their magnitude. Toxicity outbreaks were generally more intense during spring in the northern area, while in the southern area the maximum toxicity values were observed during summer. However, significant outbreaks were also recorded during autumn in CB. An important interannual variability in the location of the main PSP outbreaks was observed, with higher toxicities found in the northern area in the years 2005 and 2006 (more precisely in SJG), and then in the southern area in the years 2010 and 2011, mainly in CB and SJgeG. Regarding the interspecific variability of toxicity in species of molluscs in SJG, *A. tehuelchus* was observed to accumulate toxins for a longer time than other species, though *A. antique* registered peaks of higher toxicity.

This study highlights the relevance of PSP toxin monitoring for protecting public health and mitigating the economic impact of HAB on shellfish fisheries in Argentine Patagonia. Further research into the diversity and distribution of harmful species and their toxins, as well as the environmental conditions that lead to HAB events will help improving the current monitoring program. In addition, more interaction is needed among HAB researchers and local and state health departments at academic, community outreach, and policy levels.

## Declarations

### Author contribution statement

L. Gracia Villalobos: Analyzed and interpreted the data; Contributed reagents, materials, analysis tools or data; Wrote the paper.

Germán Marino: Performed the experiments; Contributed reagents, materials, analysis tools or data; Wrote the paper.

Norma Santinelli, Alicia Sastre: Conceived and designed the experiments; Performed the experiments.

Gastón Almandoz: Analyzed and interpreted the data; Contributed reagents, materials, analysis tools or data.

### Funding statement

This research did not receive any specific grant from funding agencies in the public, commercial, or not-for-profit sectors.

### Competing interest statement

The authors declare no conflict of interest.

### Additional information

No additional information is available for this paper.
